# Edible coatings and plasma-activated water: synergistic strategies for extending fresh produce shelf life

**DOI:** 10.3389/fnut.2026.1824743

**Published:** 2026-06-02

**Authors:** Tejaswi Boyapati, Kasiviswanathan Muthukumarappan

**Affiliations:** Agricultural and Biosystems Engineering, South Dakota State University, Brookings, SD, United States

**Keywords:** edible coating, fresh produce, hurdle technology, plasma-activated water, postharvest preservation, shelf-life extension

## Abstract

Postharvest losses of fresh fruits and vegetables constitute a global crisis, with 20–30% of products lost in rich countries and up to 55% in developing nations before reaching consumers. Although traditional chemical sanitizers and refrigeration are prevalent, the increasing demand for residue-free, clean-label preservation is stimulating interest in non-thermal options. This research critically assesses the combined use of edible coatings (ECs) and plasma-activated water (PAW) as complementary hurdle technologies for postharvest preservation of fresh foods. We contend that the sequential PAW-then-EC strategy, where PAW delivers immediate, broad-spectrum RONS-mediated microbial reduction (2–4 log CFU/g), followed by EC, creating a continuous physical and bioactive barrier, overcomes the limitations of each technology individually, in a manner no single treatment can replicate. Significant findings across several commodities (strawberries, tomatoes, apples, grapes) indicate that combination treatments prolong marketable shelf life by 40–100% compared to untreated controls, diminish enzymatic browning by inhibiting PPO and POD, and preserve firmness, color, and nutritional integrity. This study highlights significant research gaps, including the instability of reactive oxygen and nitrogen species (RONS) in lipid-rich coating matrices, the need to optimize process parameters for specific commodities, and the lack of internationally standardized methodologies for characterizing plasma-activated water (PAW). We determine that the commercial translation of EC–PAW systems necessitates scale-up engineering, lifecycle evaluation frameworks, and synchronized regulatory collaboration across principal markets.

## 1 Introduction

Fresh horticultural produce losses during postharvest are very high worldwide, reaching 20–30%, and pose a primary global concern, challenging food security and sustainability while imposing a heavy economic burden (1). In developing countries, where on-farm storage infrastructure for perishable goods is not always available, as much as 28–55% of fresh produce can be lost between harvest and consumption due to spoilage along the supply chain (2). Unlike seeds and legumes, which are durable staples, fruits and vegetables have high water activity. They are highly perishable, as moisture content promotes metabolic activity in the produce, which is sensitive to mechanical damage and microbial contamination. Post-harvest, fresh produce remains biologically active and continues to go through physiological processes such as respiration, transpiration, and enzymatic activity. These processes speed up aging, weaken texture and nutritional value, and make the food more vulnerable to microbial spoilage, leading to food loss along the supply chain from farm to table.

While traditional methods like refrigeration, chemical sanitizers, and modified atmosphere packaging still extend shelf life, they raise health and environmental concerns. As a result, there is increasing interest in non-thermal, sustainable, and environmentally friendly technologies that ensure food quality and safety while meeting consumer demand for clean-label products.

In recent years, two non-thermal, residue-free technologies a) edible coatings (3, 4) and b) plasma-activated water (PAW) (5, 6) have emerged as game-changing techniques to combat post-harvest deterioration of fresh produce. Those technologies represent two leading ways to help fresh produce stay fresher longer; they are also consistent with the ideas behind green technology and non-thermal processing. Edible coatings are thin biopolymer films applied directly to the surfaces of fresh produce. This layer acts as a partial barrier between the internal gas phase and the ambient vapor phase, thereby controlling the exchange of gases across membranes, decreasing water loss (transpiration), and the rate of respiratory gas exchange. ECs are generally produced from natural materials, such as polysaccharides, starch, cellulose, alginate, and proteins like whey, and many are enhanced with various functional compounds, including essential oils, phenolic antioxidants, and antimicrobials. Beyond maintaining micronutrient and sensory quality, the use of EC enhances appearance, mouthfeel, aroma retention, and flavor stability, as well as shelf appeal, without compromising food safety, including providing additional GRAS (Generally Recognized as Safe) statuses for food-grade components and for onsite application.

In contrast, plasma-activated water is a new method for sterilizing the product by immersing it in cold atmospheric plasma, enabling the formation of reactive oxygen and nitrogen species (RONS) such as H₂O₂, O₃, nitrites, and nitrates. Due to these species being generated, PAW exhibits potent antimicrobial and oxidative activity, making it an excellent disinfectant for inactivating various bacteria, fungi, and viruses on the surface of fresh produce and in water. In contrast, traditional chemical washes leave a residue on the surface of fresh produce. PAW is highly reactive, as it breaks down into innocuous components. Removing spoilage yeast or molds from heat-sensitive fruits and vegetables that cannot be pasteurized with hot water or cleaned is also an effective solution using PAW (7, 8).

Although ECs and PAW are effective postharvest factors that enhance quality and safety, recent research has demonstrated that combining them as a hurdle technology offers promising results (9). The researchers’ strategy is based on these complementary mechanisms: PAW provides direct microbial decontamination and enzymatic suppression, while an edible coating offers physical protection and inhibits physiological degradation. When used sequentially, typically treating with PAW followed by dipping in EC, these technologies can work together synergistically to improve safety, minimize weight loss, slow ripening and aging, and preserve texture and nutrition during extended storage (10). Studies on strawberries, apples, tomatoes, and leafy greens indicate that PAW combined with EC significantly surpasses either treatment alone, achieving over 5-log microbial reduction, increasing water-holding capacity (WHC) by up to 10%, and extending shelf life (11–14).

Despite growing evidence that EC and PAW address distinct aspects of postharvest deterioration, much of the research to date has examined them in isolation. Most evaluations focus on a single technology while only superficially addressing the others. No prior analysis has comprehensively examined the process factors, such as PAW generator type, RONS composition, drying interval between treatments, and coating formulation, that determine whether integrating these technologies yields a true synergistic effect or merely an additive one. This distinction is significant in practice: synergy means the combined treatment is more effective than either treatment alone, even at optimal dosages, whereas additivity does not imply this. This review is organized to clarify that distinction.

This review makes three contributions. First, it establishes the mechanistic rationale for why PAW and EC are complementary rather than redundant. PAW rapidly targets surface microorganisms and enzymes via RONS, while EC provides prolonged physical and bioactive protection afterward. Second, it critically assesses the process parameters that influence PAW effectiveness and coating performance, identifying the conditions under which synergy between the two technologies occurs. Third, it highlights key barriers to commercial translation, such as RONS instability in coating matrices, the absence of standardized PAW characterization protocols, and regulatory uncertainties, and suggests directions for addressing them.

## 2 Fresh produce preservation using edible coatings

Edible films and coatings are thin layers of edible substances applied to food surfaces to act as barriers against moisture, gases, or the release of soluble components. While edible coatings are safe for human consumption, synthetic plastic packaging is not. They provide renewable biopolymers, such as the resins used in this study, offering a more sustainable alternative to address the environmental issues caused by plastic waste. The coatings are designed to be semi-permeable, allowing moisture to escape and oxygen to enter. This selective permeability changes the physiological state of perishable foods and the internal microenvironment of fresh produce, helping to delay ripening and aging, maintain firmness, and reduce weight loss during storage. Edible coatings (ECs) serve as passive barriers, but unlike packaging, they can also protect by delivering functional ingredients like antimicrobial agents, antioxidants, and nutrients.

### 2.1 Composition and key properties

To enhance barrier and mechanical properties, three primary biopolymers, polysaccharides, proteins, and lipids, are commonly used in edible coatings. Polysaccharides, such as cellulose, starch, pectin, alginate, and chitosan, can form transparent, breathable films with good gas barrier properties. Notably, chitosan is biocompatible and exhibits a wide range of antimicrobial effects depending on its source (15). However, due to the high hydrophilicity and brittleness of polysaccharide films, these materials exhibit poor moisture resistance in their native form unless plasticizers or reinforcing agents, such as nanocellulose, are added. Coatings based on soy, wheat, pea, casein, and whey proteins create cohesive, flexible films that adhere well and offer good oxygen permeability. For instance, milk proteins can serve as carriers for bioactive compounds and help prevent their oxidative degradation. Nonetheless, their high-water affinity and moisture absorption can also compromise their moisture barrier properties, as with polysaccharides (16). This limitation can be addressed using lipids or enzymatic cross-linking (e.g., transglutaminase), but the pH of proteins remains a challenge. Natural waxes (such as carnauba and beeswax), fatty acids, and mono- and diglycerides serve as excellent moisture barriers, reducing transpiration and shrinkage. However, they provide poor oxygen barrier properties and low mechanical strength. On their own, they may produce an undesirable shiny or brittle surface and are often combined with polysaccharide or protein matrices to improve functionality and structure (17). Selecting the appropriate biopolymer can regulate gas and moisture barrier properties, polysaccharides and proteins slow oxidation and respiration, while lipids prevent water loss. Protein films tend to be pliable, whereas polysaccharide films require plasticizers for strength and flexibility. Blended or composite coatings offer tailored solutions: lipid-based coatings are suitable for protecting high-water-content produce, while protein-polysaccharide films are ideal for items prone to oxidation. Antimicrobial and antioxidant functionalities are increasingly incorporated into coatings using essential oils, polyphenols, or vitamins. For example, cinnamon or oregano oil destroys microbes, and ascorbic acid prevents browning. These edible films are designed to entrain bioactive agents for slow release on the produce surface, helping extend shelf life and enhance bioavailability. Table 1 provides a structured overview of the three primary biopolymer categories and composite systems used in edible coatings, comparing their key characteristics, product applications, limitations, and future development trends, including composite material innovations and nanotechnology-based approaches.

**Table 1:** Categorization of edible coatings by biopolymer source, illustrating key biopolymers and examples, principal film-forming and barrier characteristics, representative produce applications, current limitations, and future development trends, including composite material innovations and nanotechnology-based approaches.

Category	Key biopolymers/examples	Key characteristics	Produce applications	Limitations	Future trends	References
Polysaccharide-based	Chitosan, starch, alginate, cellulose (CMC, MC), pectin, carrageenan	Transparent, breathable films Good gas (O₂/CO₂) barrier Chitosan: inherent antimicrobial pH-responsive solubility (chitosan) Good film-forming ability	Strawberries, citrus, tomatoes, mango Fresh-cut produce (apples, pears) Leafy greens Stone fruits (peaches, plums)	High hydrophilicity → poor moisture barrier Brittleness without plasticizers Variable solubility across pH May require crosslinking agents	Chitosan nanoparticles for controlled release Composite with lipids to improve moisture barrier pH-responsive intelligent coatings Extraction from agri-waste (e.g., crustacean byproduct valorization)	(10, 38−40)
Protein-based	Whey protein, soy protein, casein, zein (corn), gelatin, gluten	Good O₂ barrier properties Strong mechanical films Excellent adhesion to produce surfaces Carrier for bioactive compounds Flexible when plasticized	Fresh-cut fruits (apples, peaches) Tomatoes, avocados High-respiration commodities Animal-based produce (eggs, meat)	High water affinity → moisture absorption Sensitive to pH and heat Allergen concerns (milk, soy, wheat) Higher cost than polysaccharides	Enzymatic crosslinking (transglutaminase) to improve WVP Plant-protein composites for allergen-free options Whey protein isolate nanostructures Use of upcycled food-processing protein streams	(10, 13, 40)
Lipid-based	Carnauba wax, beeswax, candelilla wax, stearic acid, oleic acid, monoglycerides	Excellent moisture barrier (hydrophobic) Reduces transpiration effectively Imparts glossy appearance Low water vapor permeability	Citrus fruits, apples, cucumbers Root vegetables (carrots, potatoes) Tropical fruits (mango, avocado) High-moisture produce	Poor gas barrier (O₂/CO₂) Low mechanical strength alone May cause anaerobic conditions if over-applied Can produce off-flavors at high concentrations	Nanoemulsion systems for uniform application Combination with polysaccharides (bilayer coatings) Plant-oil-based functional coatings (essential oils) Encapsulation of PUFA for nutritional enhancement	(10, 41, 42)
Composite/multi-layer	Chitosan + alginate; whey protein + wax; starch + lipid; protein-polysaccharide bilayers	Tailored gas and moisture barriers Enhanced mechanical integrity Synergistic antimicrobial/antioxidant effects Customizable release profiles	Fresh-cut salads and mixed produce High-value export commodities Minimally processed fruits and vegetables Temperature-sensitive produce	Complex formulation and manufacturing Higher cost and scale-up challenges Requires optimization per commodity Regulatory complexity for multi-component systems	3D-printed edible coatings for precision deposition Smart/active coatings with spoilage indicators AI-driven formulation optimization Integration with PAW (PAW as aqueous phase in coating solution)	(10–12, 43)

CMC, carboxymethyl cellulose; MC, methylcellulose; WVP, water vapor permeability; OTR, oxygen transmission rate; PUFA, polyunsaturated fatty acids.

### 2.2 Application methods

Edible coatings are easy to apply and suitable for both laboratory and industrial use. The two primary methods are dipping and spraying. Dipping provides complete coverage and a uniform coating, but may require multiple applications and drying time. Spraying uses mist or air-blast systems, making it efficient and ideal for high-volume production and delicate fruits. The choice of method depends on the type of produce and coating viscosity; emulsions are used for spraying, while thicker polysaccharide solutions are used for dipping.

However, methods applied on a smaller scale or only for experimental purposes (brushing/spreading) are also available. Nanofibrous or ultra-thin, uniform coatings have been developed using advanced methods, such as electrostatic spraying. For instance, the shelf life of guava has been improved using electro-sprayed emulsions to reduce weight loss and microbial spoilage (18). After application, the surface must be dried; this can be done with an ambient- or forced-air dryer. Technologies such as infrared or vacuum-assisted drying are being developed to accelerate film formation without damaging the green material.

Edible films are usually pre-formed and applied to food items or between them, whereas coatings form directly on the food during drying. Fresh produce applications are more relevant and highly tested in coatings.

### 2.3 Impact on quality and shelf life

Edible coatings affect produce quality primarily by altering its initial environment and forming a protective barrier against gases. Studies have shown that coatings reduced weight loss, delayed softening, and controlled microbial growth. The quality, value, and shelf life of foods are increased with edible coatings compared to those without treatments. For example, tomatoes coated with a composite gelatine–chitosan–starch coating maintained higher firmness and stable pH values throughout their storage time compared to uncoated controls (19).

The coatings lower the rate of respiration and consumption of organic acids, delaying the pH increase at ripening. Because of this lower metabolic activity, less cell wall degradation occurs, and so the firmness is better retained. Coatings also create a partial modified atmosphere with higher CO₂ and lower O₂ levels, which, in turn, downregulate the ethylene response, thus retarding the onset of symptoms such as color break and softening. Good results in maintaining mango (20) freshness have also been obtained and tomatoes (21) when they are coated with chitosan, which helps to keep ripening at bay for more than a week.

Coatings act as physical barriers to restrict access to cell surfaces and dangerous molecules, and can also serve as antimicrobial delivery systems. Chitosan is an antimicrobial compound, while essential oils are used as additives in coatings (such as oregano or clove oil). These antimicrobial additives reduce levels of spoilage bacteria and fungi on the fruit surface. Coatings also make wood surfaces drier and reduce their oxygen content, creating conditions unfavorable for the growth of microorganisms.

This process generally results in a substantial increase in shelf life. Several studies reveal that the storage period is extended 1.5–2-fold by ECs under controlled conditions of storage (22). For example, when compared to untreated samples, coated tomatoes remain marketable for 15–20 days rather than 10 days. Strawberries, citrus like oranges and tangerines, and even papayas are reaping benefits from this. In fresh-cut fruits such as apples and pears, the incorporation of different anti-browning agents through integrated coatings helps extend their shelf life by delaying discoloration and keeping a fresher appearance.

Nutritionally, edible coatings are used to maintain sensitive compounds, such as vitamin C and phenolics, by reducing oxidative and enzymatic degradation. For instance, *aloe vera* and pectin-based coatings have exhibited higher accumulation of vitamin C in the coated mangoes and guavas over time (23, 24). Usually, the sensory attributes are maintained, and the edible coating improves appearance by making the food glossy. In general, most properly manufactured coatings will have no taste or odor. However, this is not always the case, depending on how the extracts or essential oils are added to affect the coating.

### 2.4 Limitations

Edible coatings offer some advantages in preserving fresh produce, but they also have limitations. They work best with freshly harvested items and are not intended to reverse spoilage or sterilize surfaces. Coatings can become a liability if they trap bacteria, allowing microorganisms to persist and go undetected without proper sanitation. This balance is crucial because over-sealed coatings can produce off-flavors, while undersized-sealed coatings fail to protect the product. Flavors, essential oils, and other flavor additives should be sensory evaluated when used. Even if coatings are inexpensive and some natural biopolymers are GRAS-compliant, their application still involves a postharvest step. However, when combined with proper hygiene and storage practices, they help improve food quality retention, prevent waste, and offer a sustainable preservation option.

## 3 Produce sanitation and shelf-life extension using plasma-activated water

Efforts to combat spoilage and limit losses of fresh produce after harvest reveal that the food industry is seeking more efficient sanitization strategies, along with innovations in packaging and the development of edible coatings. Although traditional chemical disinfectants such as chlorine-based washes are still widely used, they have some limitations, including potential for harmful residues, increasing consumer reticence on the use of chemicals due to their lingering presence on fresh produce, and reaching Europe’s tight limits of detection (≤10 ppb) (25); thereby reducing the effect on biofilms. Within this context, plasma-activated water (PAW) was developed as a non-thermal, non-chemical antimicrobial agent for the treatment of fresh produce. PAW is generated by the treatment of water with cold atmospheric plasma (CAP) and is enriched with reactive oxygen and nitrogen species (RONS). These reactive species are highly antimicrobial, broad-spectrum, and capable of killing all microbes tested so far, and they decompose into non-toxic byproducts such as water, oxygen, and nitrate. These important attributes leave no dangerous residues on the produce treated. This section addresses the generation, physicochemical properties, antimicrobial mechanisms, and practical applications of PAW in improving the quality and safety of fresh produce.

### 3.1 Generation and properties of PAW

Plasma is what we all think of as the fourth state of matter after solids, liquids, and gases, where it is partially or fully ionized and made up of free electrons, positive (and negative) ions, and neutral particles in an excited state (54). This occurs when electrical power is passed through a gas, ionizing it via collisions between high-energy electrons and gas molecules. Depending on temperature and thermodynamic equilibrium, plasma is well categorized into thermal (hot) and non-thermal (cold) plasmas (CP). Traditional thermal plasma runs in an equilibrium environment where temperature can reach 4,000–20,000 K, but CP runs in a non-equilibrium regime at much lower temperatures (100–300 K) with enhanced electron density (~109 cm − 3), and it can efficiently generate reactive species without causing any thermal damage; thereby making it a suitable candidate for food processing and decontamination applications (26). Currently, CP can be generated through several methods, such as dielectric barrier discharge (DBD), corona discharge, microwave discharge (MD), gliding arc (GA), and atmospheric pressure plasma jet (APPJ). Both approaches have their distinct advantages in scalability, energy efficiency, and application flexibility (26). In addition, plasma generation techniques are generally divided by operating pressure (low or atmospheric) and by exposure type (direct, semi-direct, or indirect), depending on the application, such as microbial inactivation or surface treatment.

Cold plasma (CP)^1^ technology has been used to develop several emerging applications; one of the most important is plasma-activated water (PAW), which is generated by directly or indirectly immersing CP in water. In this process, plasma generated in the gas phase is bubbled into or applied to water, resulting in reactive oxygen and nitrogen species (RONS) like peroxide (H_2_O_2_), ozone (O_3_), nitrate (NO_3_ −), nitrite (NO_2_−) hydroxyl radicals(·OH) and (ONOO −) dissolving in an aqueous solution (5, 44). The next step is a PAW with low pH (2–4), high Oxidation–reduction potential (ORP, often above +600 mV), and high conductivity, indicating strong oxidizing properties. The ORP is significantly affected by the stable species, such as hydrogen peroxide, and nitrite contributes to the formation of peroxy nitrous or peroxy nitric acids at acidic pH. By adjusting plasma source parameters, the composition and reactivity of PAW can be tuned, allowing its synthesis to change the balance of reactive species. Oxygen-rich feed gases promote the production of reactive nitrogen species (RNS) while the presence of nitrogen-containing gases stimulates the formation of reactive oxygen species (ROS). Their tunability may make PAW a viable non-residue alternative to traditional liquid chemical treatments, making it a significant, practically feasible technology for food safety, agriculture, and disinfection.

#### 3.1.1 Process parameters and RONS tunability

The antimicrobial and preservation effectiveness of PAW is not a fixed characteristic; rather, it is a variable outcome influenced by the interplay among plasma generator design, operational parameters, and water chemistry. It is important to understand these interactions to make PAW work best for certain types of fresh produce and to make sense of the wide range of effectiveness reported across studies.

##### 3.1.1.1 Feed gas composition

The composition of the feed gas is the most important factor that determines which reactive species are most common in PAW. When air is used as the feed gas, both reactive oxygen species (ROS) and reactive nitrogen species (RNS) are generated simultaneously. This makes a balanced mix of H₂O₂, NO₂^−^, NO₃^−^, and trace amounts of OH• and O₃. Air is the most common choice for food applications because it does not need a separate gas supply system and has broad-spectrum antimicrobial activity. Oxygen-rich feed gases promote the production of reactive oxygen species (ROS), particularly H₂O₂ and O₃, while the presence of nitrogen-containing gases stimulates the formation of reactive nitrogen species (RNS), particularly NO₂^−^ and NO₃^−^. These compounds lower the pH of PAW and may be especially useful for enzyme inhibition applications, such as stopping PPO from browning fresh-cut fruit. Noble gases like argon and helium make very energetic but mostly short-lived particles. They are used in basic research, but they are too expensive for food preparation. A significant point is that several published studies on PAW performance fail to provide accurate details on feed gas composition, rendering cross-study comparisons meaningless. Subsequent studies must implement consistent reporting that includes feed gas identity, purity, and flow rate as essential parameters of PAW-generating settings (6, 27).

##### 3.1.1.2 Plasma generator design and configuration

The three main ways to make plasma that are employed in food-related PAW research, dielectric barrier discharge (DBD), atmospheric pressure plasma jet (APPJ), and gliding arc discharge, are not all equally good for usage on fresh produce (Table 2). DBD is the most common setup in food science because it operates at standard air pressure, does not require specialized gas handling, can handle large treatment volumes, and is already used in industry for surface treatment and ozone generation. In DBD systems, the distance between the electrodes and the dielectric material both affect how evenly the discharge occurs and how much energy is delivered to each unit of water. This, in turn, affects the density of RONS and the composition of PAW. APPJ makes a focused, high-energy plasma jet that works well for small-area precision treatment or for directly activating coating solutions. However, its small treatment area makes it hard to use for washing large amounts of goods. Gliding arc systems operate at higher power densities and produce RONS faster, but the heat they generate makes them less suitable for heat-sensitive goods. A significant design feature sometimes overlooked in the food literature is the distinction between direct and indirect treatments. In direct treatment, the plasma discharge directly contacts the produce, adding UV photons and electric-field effects to the RONS. Plasma is initially applied to water in indirect treatment, which is what PAW stands for. The resulting activated liquid then comes into contact with the produce. Indirect therapy gives you much more control over how much medicine you get, does not damage surfaces with UV light, and is easier to standardize in factories (26, 28).

**Table 2:** Comparison of plasma generation methods used for PAW production in fresh produce applications, including dielectric barrier discharge (DBD), atmospheric pressure plasma jet (APPJ), and gliding arc discharge.

Feature	DBD	APPJ	Gliding Arc
Operating pressure	Atmospheric	Atmospheric	Atmospheric
Treatment scale	Large volume (bulk wash)	Small area/precise	Medium–large volume
Primary RONS generated	H₂O₂, NO₂^−^, NO₃^−^, O₃	OH•, O•, O₃	NO•, NO₂^−^, H₂O₂
Thermal effects on produce	Minimal	Minimal	Moderate
Scalability for industry	High	Low–moderate	Moderate
Feed gas flexibility	High	Moderate	Moderate
Best suited for	Bulk PAW generation for dipping/washing	Coating solution activation, surface jets	Rapid high-concentration PAW generation
Key limitation	RONS uniformity depends on gap geometry	Limited coverage area	Higher operating temperature
References	(27, 45)	(46)	(6, 47)

Parameters compared include operating pressure, treatment scale, primary reactive oxygen and nitrogen species (RONS) generated, thermal effects on produce, industrial scalability, feed gas flexibility, optimal application context, and key limitations. DBD, dielectric barrier discharge; APPJ, atmospheric pressure plasma jet; RONS, reactive oxygen and nitrogen species.

##### 3.1.1.3 Treatment time and water volume

The treatment period and the volume of water being activated both affect the effective RONS concentration in PAW. There is no clear relationship between treatment time and RONS accumulation. Long-lived molecules like H₂O₂, NO₂^−^, and NO₃^−^ continue to accumulate as the treatment progresses, though the rate of accumulation slows after about 15–20 min in most DBD setups because reactions that break them down become more important (27). Short-lived species like OH•, O₂•^−^, and singlet oxygen quickly reach a pseudo-steady state and do not build up over time. This means that treating long-lived species for too long can lead to over-activation, which can cause surface oxidation, tissue softening, or off-flavor development in foods sensitive to these changes. There is an inverse relationship between water volume and RONS concentration: activating a smaller volume of water under the same plasma conditions results in a higher concentration of PAW. This means that treatment time alone does not provide a complete picture of PAW exposure, and results from studies that used different water volumes cannot be directly compared unless RONS concentrations are examined separately. The quality of the water at the start also changes the composition of PAW. The conductivity, mineral content, and initial pH all affect how RONS dissolve in water and how quickly they do so. When distilled or deionized water is used instead of tap water, the PAW is different. In published food science studies, this variable is rarely controlled for or reported (6). It is suggested that subsequent research utilize a PAW dose concept similar to the concentration × time (CT) model employed in traditional chemical sanitation, represented as a function of the measured H₂O₂ concentration, contact duration, and treatment temperature (27).

##### 3.1.1.4 RONS stability and the practical treatment window

One important but often-ignored part of PAW for preserving food is how stable its reactive components are over time after they are made. The half-lives of reactive species in PAW vary widely, which affects how PAW is stored, moved, and used. Some short-lived organisms that exist for only a few microseconds to a few milliseconds in water include OH•, O₂•^−^, and singlet oxygen. They are mostly to blame for killing germs where they are made (26). By the time PAW is transferred to a dipping tank or spray system in a practical processing setting, these species are essentially depleted. Long-lived species principally H₂O₂ and NO₂^−^ persist for hours to days, depending on storage conditions, and are responsible for the residual antimicrobial effects observed in treated produce during subsequent refrigerated storage, such as the continued pathogen reduction reported in PAW-treated strawberries (29). High temperatures, light, and transition-metal ions accelerate the breakdown of H₂O₂. On the other hand, NO₂^−^ stays stable when stored in the dark and at low temperatures (6). Based on current evidence, PAW should be applied within a few hours of generation to ensure adequate H₂O₂ activity, stored cold and in sealed, light-opaque containers if a delay is unavoidable, and characterized at the point of application rather than only at the point of generation. Reporting PAW age at the time of application should be adopted as a standard practice in the literature, as this variable significantly influences measured efficacy but is almost never disclosed (27, 30).

##### 3.1.1.5 pH and ORP as practical quality control indicators

The pH and oxidation–reduction potential (ORP) of PAW can be determined with conventional benchtop meters that any food processing plant should have. You do not need specialized equipment, such as fluorescence probes or chemiluminescence assays, to detect RONS in real time. If the pH of PAW is above about 5.0 it is probably not sufficiently activated to work as an antibacterial agent and should be regarded as out of specification. On the other hand, PAW with a pH below about 2.5 can cause surface acidification in contact-sensitive products such as berries and leafy greens. This can speed up tissue softening, change the stability of anthocyanins, and change the surface color in ways that make the goods less appealing to buyers. It is necessary to calibrate the ideal pH and ORP range for a certain product. Current research shows that a pH range of 2.5–4.0 and an ORP level of 500 mV or above is a good goal for most fresh produce uses while reducing the risk of quality loss (5, 6, 27). Setting pH and ORP limits for each type of commodity as go/no-go quality control standards for PAW in businesses is a realistic and doable short-term goal for standardization that the field should focus on. Using this methodology would also make it much easier to reproduce and compare published PAW efficacy data. Right now, the data is very different because treatment intensity is characterized in terms of generator settings instead of measurable liquid-phase parameters (27, 31).

##### 3.1.1.6 RONS stability within coating matrices

**Figure 1:**
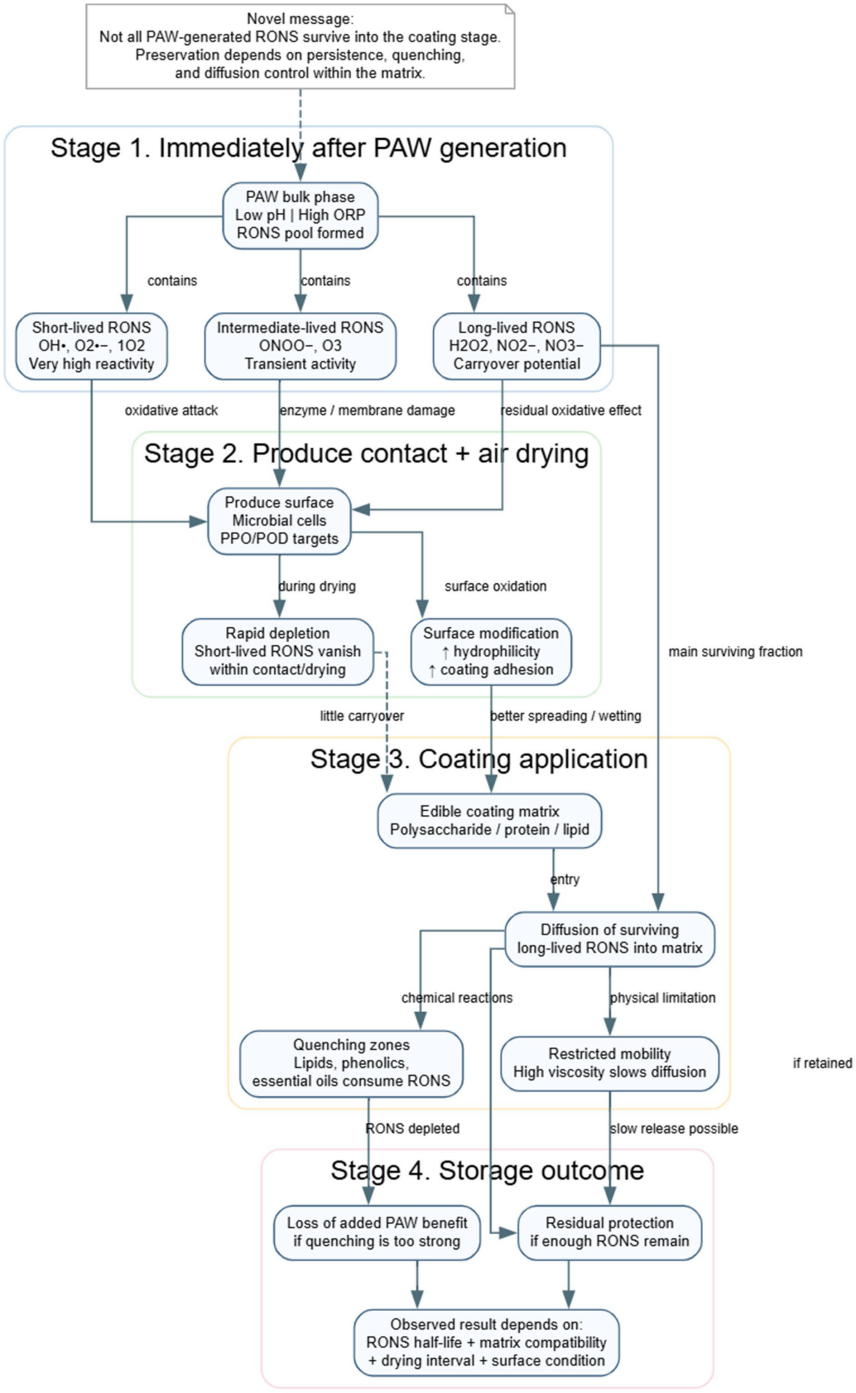
RONS fate and decay in the PAW-EC system. The figure illustrates the temporal evolution of reactive oxygen and nitrogen species (RONS) from plasma-activated water (PAW) generation through to the edible coating (EC) application stage. Short-lived species—including hydroxyl radical (OH•), superoxide radical anion (O_2_•-), and singlet oxygen (^1^O_2_)—have aqueous half-lives of microseconds to milliseconds and are effectively depleted before PAW reaches the produce surface in any practical processing setting. Long-lived species—principally hydrogen peroxide (H_2_O_2_) and nitrite (NO_2_-)—persist for hours to days under cold, dark, sealed storage conditions and are responsible for the residual antimicrobial activity observed in PAW-treated produce during refrigerated storage. Once an edible coating is applied, lipid components, phenolic antioxidants, and essential oil additives within the coating matrix rapidly quench remaining H_2_O_2_ through competitive oxidation reactions, potentially eliminating antimicrobial activity before the coating contacts the produce surface. High-viscosity polysaccharide matrices further impede RONS diffusion to the produce interface. These interactions have not been systematically characterized for commonly used coating systems, which represents a critical gap in PAW-EC integration research. ORP, oxidation–reduction potential.

When PAW is added directly to an edible coating formulation, as is increasingly common (see section 4.2), the stability of RONS in the coating matrix becomes a key consideration. Common ingredients in functional coating formulations, such as lipid components, phenolic antioxidants, and essential oil additives, can rapidly quench H₂O₂ and other reactive species through competitive oxidation reactions. This could negate the antimicrobial benefit of adding PAW before the coating is even applied to the produce surface (12). High-viscosity polysaccharide matrices may also impede RONS diffusion and reduce their effective concentration at the coating–produce interface. These interactions have not yet been systematically characterized for the coating systems most commonly used in fresh produce applications, representing one of the most important open questions in PAW–EC integration research. Until RONS half-lives and residual antimicrobial activity are characterized within representative coating formulations under realistic processing conditions, claims about the antimicrobial activity of PAW-incorporated coatings should be interpreted with caution (12, 43). Despite frequent claims of synergistic effects, there are no clear criteria for distinguishing synergy from additive responses. Figure 1 presents a decision framework for classifying combined PAW–EC outcomes. This framework highlights that only a fraction of long-lived RONS persists into the coating stage, and their effectiveness depends on matrix compatibility, diffusion constraints, and quenching reactions.

### 3.2 Mechanisms of microbial inactivation

Cold plasma (CP) treatment is an innovative non-thermal method for microbial decontamination that uses reactive species generated at low temperatures to eliminate pathogens. Despite extensive studies on its antimicrobial efficacy, the exact mechanisms remain partially understood. Multiple synergistic mechanisms contribute to microbial inactivation by CP, including damage to the phospholipid membrane and DNA, disruption of intracellular contents, protein oxidation, and the formation of cytotoxic compounds (26, 32).

CP primarily targets the bacterial cell membrane. Phospholipids interact with reactive species [atomic oxygen, ozone, hydroxyl radical (OH•), superoxide (O_2_−•)], leading to lipid peroxidation. This leads to pore formation, increased permeability, and intracellular leakage, resulting in morphological changes and eventually cell lysis (49). Additionally, oxidative stress or damage, along with mechanical damage from electroporation or chemical oxidation by reactive oxygen species (ROS), weakens the membrane. Oxidative degradation is evidenced by markers such as malondialdehyde (MDA) and 4-hydroxynonenal (4-HNE).

Outside the membrane, CP species penetrate cells and damage intracellular components. Among types of DNA damage are oxidative modifications (indicated by the presence of 8-OHdG), strand breaks, and overwhelmed repair systems. Protein oxidation involves changes in amino acid residues and structural denaturation, which can impair the functions of enzymes vital to cellular metabolism (26). It can also inactivate key enzymes crucial for food safety and quality, such as polyphenol oxidase (PPO) and peroxidase (POD), thereby affecting microbial viability. It is also essential to note that CP indirectly facilitates chemical reactions, such as the Haber-Weiss reaction, in which iron catalyzes the formation of hydroxyl radicals. Additionally, nitric oxide (NO) can generate cytotoxic species, such as peroxynitrite (ONOO^−^) and nitrogen dioxide (NO₂), which further contribute to oxidative damage.

This broad spectrum of action likely explains CP’s effectiveness against both bacteria and fungi, killing them by targeting cell membranes, inducing DNA fragmentation and protein denaturation, and inducing apoptosis in eukaryotic cells. Mitochondria appear to play a role in apoptosis-induced cell death, which is important for perioperative diagnosis as it compromises mitochondrial function, induces subsequent ROS overproduction, and permits trans-epithelial penetration of CP (26).

Furthermore, plasma-activated water (PAW) also exhibits antimicrobial properties through oxidative pathways (48). With reactive oxygen and nitrogen species (RONS) such as H_2_O_2_, OH•, NO, and ONOO^−^, PAW disrupts the cell membrane and destroys enzymatic activity, leading to DNA damage. The high acidity (pH 2–4) and oxidation–reduction potential (ORP) of PAW have been shown to enhance its microbicidal effectiveness. Its three-pronged mechanism of oxidative stress, structural disruption, and enzymatic inhibition indicates that PAW is a highly effective antimicrobial suitable for use as a sanitizer in food environments.

Thus, CP or PAW exhibit robust antimicrobial activity that circumvents resistance mechanisms employed by common sanitizers, indicating their promising applications in food safety and preservation.

### 3.3 Efficacy on fresh produce

A recent study demonstrated substantial, broad-spectrum antimicrobial efficacy of PAW across a variety of fresh produce commodities. Natural microflora and inoculated pathogens have been reduced by 1–3 log₁₀ CFU/g when short exposure times were utilized in a study by Ma et al. (33) A 5-min PAW wash on strawberries resulted in 2 log reductions in *Staphylococcus aureus* levels immediately after treatment and up to 4 log reductions during refrigerated storage, suggesting the presence of residual antimicrobial effects due to any remaining reactive species. PAW is effective in reducing *Escherichia coli*, *Listeria monocytogenes*, *Salmonella* spp., and fungal pathogens (*Penicillium* spp.) on citrus and other fruits (34).

In addition to microbial inactivation, PAW has been observed to affect the activity of enzymes that degrade food quality. For example, Perinban et al. (35) showed that PAW significantly suppressed PPO activity in fresh-cut apple slices over 12 days, leading to reduced browning and higher phenolic content. Then, after slight activation (presumably a stress response) of peroxidase (POD), enzymatic browning was significantly suppressed. The effect is primarily ascribed to oxidation, modification, and denaturation of the enzyme active sites, as well as acidity in PAW.

Crucially, when used at the proper time and at the proper application level, PAW does not harm a product’s sensory or nutritional quality. Many studies have indicated that texture, color, and pH were not affected by PAW treatment. In contrast, a few other studies reported changes in these qualitative traits after PAW use. Strawberries treated with PAW did not affect firmness or visual quality, such as those used by Ma at al. (33). Apple slices treated with PAW also had better firmness and less browning than the control group. *In vitro* results show a reduction in firmness beyond 6 min of exposure due to central activation or over-oxidation, leading to tissue melting with prolonged application; this can be prevented by carefully selecting the treatment time. Typical mild activation times (10–20 min for water treatment) and short product exposure times (5–10 min) are usually long enough to accomplish microbicidal reduction while preserving quality. However, PAW has been linked to positive metabolic responses in treated vegetables, which is not surprising. Exposure to PAW increased phenolic content in cherry tomatoes during storage, indicating a hermetic response that would improve antioxidant properties. Table 3 summarizes recent literature on PAW efficacy across multiple fresh produce commodities, highlighting the diversity of treatment conditions, target pathogens, microbial reduction levels achieved, and associated quality outcomes.

**Table 3:** Summary of recent literature on plasma-activated water (PAW) efficacy on fresh produce, including treatment conditions, target microorganisms or quality endpoints, level of microbial reduction achieved, and effects on produce quality attributes.

Produce	Pathogen/target	PAW conditions	Microbial reduction	Quality outcomes	References
Strawberries	*S. aureus*, natural microflora	5-min wash; DBD plasma	2 log (immediate); up to 4 log during refrigerated storage	No change in firmness or visual quality; residual antimicrobial effect observed	(33)
Fresh-cut apple slices	PPO/POD enzymes; natural microflora	10–20 min PAW treatment	1.05 log (aerobic count) over 12 days	Reduced browning; higher phenolic content; better firmness vs. control	(35)
Cherry tomatoes	Bacteria, fungi	PAW + WOLPE (PCW)	2.11 log (bacteria); 3.99 log (fungi)	Delayed softening; maintained color, pH, antioxidants; 14 days at 4 °C	(43)
Cucamelons/cucurbits	*E. coli*, *Salmonella*, *L. monocytogenes*	2-min in-situ DBD, 1,500 Hz	3 log CFU/g reduction	No negative impact on quality or shelf life vs. NaOCl which reduced shelf life	(50)
Mandarin (cv. Orlando Tangelo)	Natural microflora; oxidative stress	PAW immersion 2–4 min; 5°C storage	Reduced microbial load and lipid peroxidation	Improved vitamin C, TSS, antioxidant enzymes; extended shelf life 45 days	(51)
Red grapes	Bacteria, yeasts, coliforms	PAW + chitosan + ascorbic acid coating	~2.62 log (bacteria); 1.72 log (yeasts/molds); 1.1 log (coliforms)	Boosted phenols (~111.2 mg GAE/100 g); extended ≥8 days at 4 °C	(43)
Fresh-cut kiwifruit	*S. aureus*	PAW treatment	~1.80 log CFU/g reduction	Maintained sensory attributes	(34)

DBD, dielectric barrier discharge; PPO, polyphenol oxidase; POD, peroxidase; TSS, total soluble solids; CFU, colony-forming units.

### 3.4 Advantages and limitations

Plasma Activated Water (PAW) is an exciting new food sanitizing technology with several key benefits over chemical sanitizing systems. The PAW process produces reactive species that naturally break down to harmless byproducts, water, oxygen, and safe concentrations of nitrates and nitrites naturally occurring in produce. PAW has the advantage of not forming destructive chlorinated byproducts, and it is not narrow-spectrum, as it is effective against pathogens within biofilms and on surface roughness features.

Due to the technology’s multi-target activity, it is challenging for microorganisms to develop resistance. PAW generators need air, electricity, and water; thus, they are simple, affordable, and environmentally friendly, with the advantage of not requiring the handling or storage of hazardous chemicals.

Nonetheless, PAW has downsides. The desired >5 log₁₀ reductions could be achieved with longer treatment times or gentle treatments such as ultrasound or vortexing (36). The reactive species do not last long, so new PAW generations or periodic replenishment are needed within continuous processing systems (30). There are scalability issues with high-throughput manufacturing lines, and uniform contact with the surfaces is necessary to achieve effective sanitation.

Since PAW provides no later antimicrobial protection after reactive species breakdown, its combination with secondary treatments, such as edible coatings, could provide simultaneous immediate microbial reduction and longer-term storage protection (Table 4).

**Table 4:** Synergistic mechanisms and case studies of PAW and edible coating (EC) hurdle technology organized by mechanism category.

Mechanism category	PAW treatment (hurdle 1)	Edible coating (hurdle 2)	Synergistic effect	References
Microbial control	Immediate action: RONS attack on cell membranes Oxidative damage to proteins/DNA pH stress (2.5–4.5) High ORP (800–1,200 mV) Rapid microbial kill	Sustained protection: Physical barrier against recontamination Active compound release Antimicrobial agents (essential oils) Prevention of cross-contamination Long-term microbial control	Enhanced efficacy: Sublethal PAW damage increases susceptibility Coating prevents recovery/recontamination Continuous antimicrobial pressure Synergistic Index > 1.0	(13, 27, 50)
Quality preservation	Enzyme suppression: PPO inactivation (prevents browning) POD suppression Oxidative enzyme inhibition Extends lag phase of deterioration	Physical protection: Moisture loss reduction Gas exchange control (O₂/CO₂) Respiration rate modulation Texture maintenance (turgidity) Antioxidant delivery	Comprehensive protection: Initial enzymes kill + barrier protection Delayed senescence onset Enhanced color/texture retention Quality retention > 80%	(9, 11, 12)
Surface modification	Surface enhancement: Increased hydrophilicity Oxidative surface modification Improved wettability Enhanced surface energy Contaminant removal	Improved adhesion: Better coating spreading Film formation Uniform coverage Stronger adhesion Reduced coating defects	Optimized coating: Superior film uniformity Enhanced barrier properties Improved mechanical integrity Better overall coating performance	(11, 12)
Storage stability	Initial decontamination: Reduced surface microbial load Extended lag phase Lower spoilage enzyme activity Delayed deterioration onset	Long-term protection: Continued microbial suppression Controlled atmosphere Sustained active compound release	Extended shelf life: Significant storage extension Maintained marketable quality Reduced postharvest losses 40–100% shelf-life increase	(10, 13, 50)

For each mechanism, the table identifies the specific PAW contribution (Hurdle 1), the specific EC contribution (Hurdle 2), the synergistic effect achieved when both are combined, and the supporting references. The table demonstrates how PAW and EC address distinct aspects of postharvest deterioration—microbial inactivation, enzymatic browning, moisture loss, and respiration—such that their combined action exceeds the sum of individual contributions. PAW, plasma-activated water; EC, edible coating; RONS, reactive oxygen and nitrogen species; PPO, polyphenol oxidase; POD, peroxidase.

## 4 Plasma-activated water and edible coatings together

Both edible coatings and plasma-activated water (PAW) target different aspects of postharvest deterioration. ECs are designed to delay the physicochemical degradation of a product (moisture loss, respiration, ripening, and oxidative changes). In contrast, PAW is more appropriate for countering microbial spoilage and enzymatic spoilage. Assembly of the PBNSP (Polysaccharide-Based Natural Structured Polymer) and CTG (Clove Essential Oil-Loaded Tetrahydrocurcumin Gel or Coating) a sequential dual-treatment approach, which, when combined, have been proposed in Wang and Wu (37) to act as a hurdle technology to improve the preservation of fresh-cut salad products.

**Figure 2:**
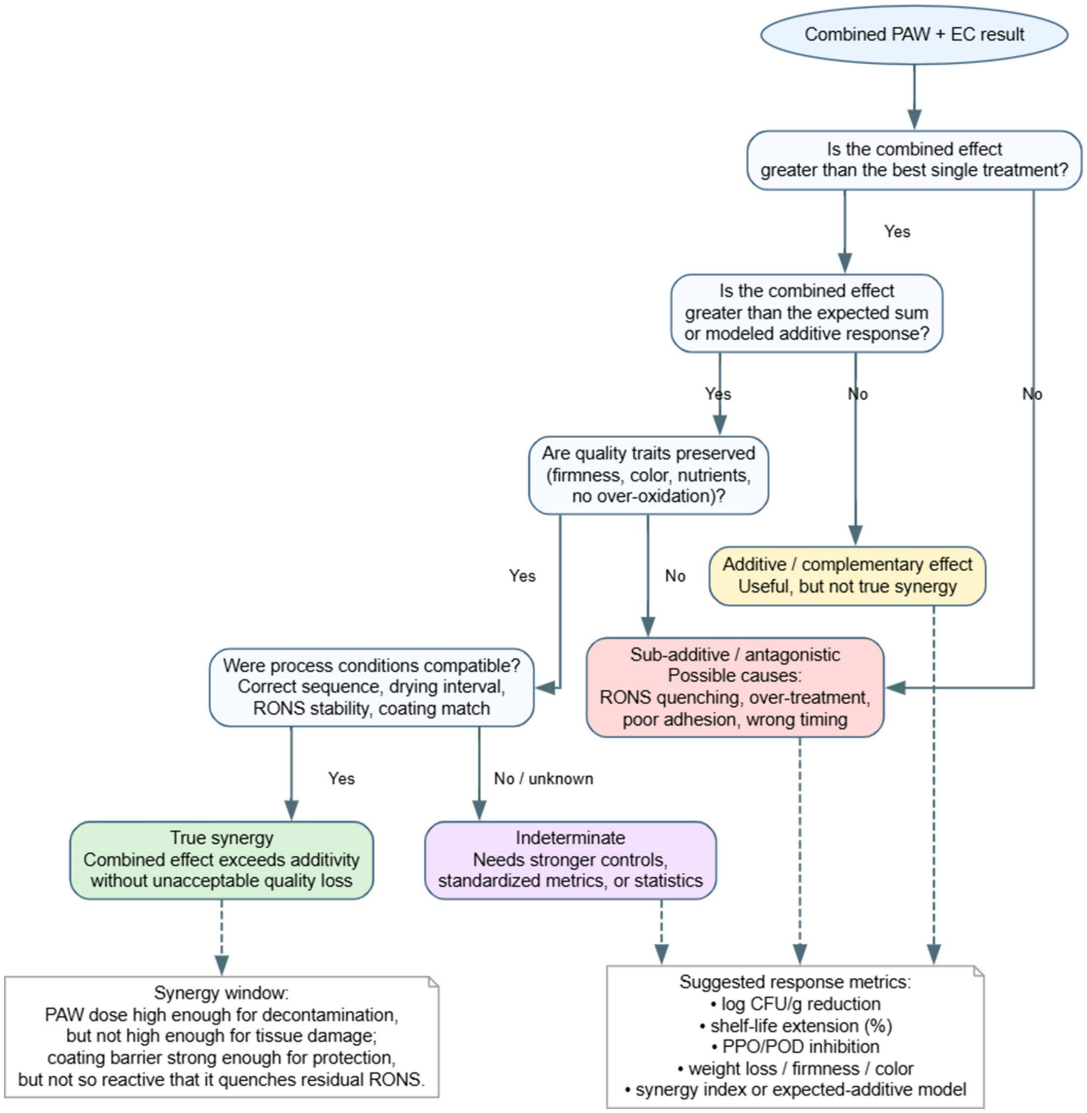
Synergy versus additivity decision framework for combined PAW + EC treatments. The framework provides a structured approach for classifying the outcome of combined plasma-activated water (PAW) and edible coating (EC) treatments as synergistic (synergistic index SI > 1.25), additive (SI ≈ 1.0–1.25), or sub-additive (SI < 1.0). Classification proceeds through four sequential decision nodes: (1) Was the correct treatment sequence followed, with PAW applied before EC? (2) Was PAW applied within its effective activity window, specifically within a few hours of generation and at pH 2.5–4.0 with ORP above 500 mV? (3) Is the coating matrix compatible with RONS persistence, meaning free from lipid components or antioxidant additives that would rapidly quench H_2_O_2_? (4) Does the measured synergistic index exceed 1.25? Studies that satisfy all four conditions are expected to fall in the synergistic zone. Studies reporting combined PAW-EC outcomes in cherry tomatoes (13, 43), Granny Smith apples (11), and red grapes (43) are mapped onto the framework; most fall within the additive-to-synergistic transition zone. SI, synergistic index, defined as the ratio of the combined treatment effect to the sum of individual treatment effects measured under identical conditions. RONS, reactive oxygen and nitrogen species.

The more usual practice is as the preceding sterilizing step in a sequence (e.g., first to sanitize by killing surface microorganisms and enzymes), when steaming is not possible. This is then covered with an edible coating, which acts as a physical barrier to moisture and oxygen, hopefully helping to extend the action of PAW by sequestering reactive species at the surface and reducing re-contamination. In addition, coatings can be tailored to incorporate antimicrobial or antiradical agents, thereby extending the preservation period. However, this sequence also plays to the strengths of each method: PAW delivers immediate antimicrobial activity, while the edible coating provides passive protection over time. As shown in Table 5, the combination of PAW and edible coatings significantly improved postharvest outcomes in multiple commodities. All studies reported increased microbial reduction, delayed spoilage, and preservation of sensory and nutritional quality, with synergistic indices exceeding 1.25. Despite frequent claims of synergistic effects, there are no clear criteria for distinguishing synergy from additive responses. Figure 2 presents a decision framework for classifying combined PAW–EC outcomes.

**Table 5:** Summary of recent studies on the combined application of cold plasma or plasma-activated water (PAW) and edible coatings for postharvest preservation of fresh produce, including produce commodity, plasma treatment conditions, edible coating formulation, key findings on microbial reduction and quality outcomes, and references.

Produce/product	Plasma treatment	Edible coating	Key findings	References
Cherry tomatoes	Plasma-activated water (PAW)	Welsh onion leaf protein extract (WOLPE)	PAW + 5% WOLPE (PCW) reduced bacteria by 2.11 log CFU/g and fungi by 3.99 log CFU/g; delayed softening; maintained color, pH, antioxidant capacity, sensory and nutritional quality; extended shelf life at 4 °C for 14 days.	(43)
Cherry tomatoes	Plasma-activated water (PAW)	Sodium caseinate–guar gum–beeswax coating	Maintained color, pH, weight, and firmness over 14 days at 4 °C; boosted antioxidants; eliminated yeasts/molds, Enterobacteriaceae, and Pseudomonas; reduced total bacteria by ~2 log CFU/cm^2^.	(13)
Granny Smith apples	Cold plasma (pre-coating)	Sodium alginate coating	Plasma-activated coating reduced weight loss, preserved firmness, titratable acidity, and color; lowered microbial load; SEM showed more uniform coating coverage.	(11)
Various fresh produce (review)	Cold plasma activation	Various edible coatings	Plasma activation improves coating mechanical and barrier properties; enhances fresh produce quality and aligns with sustainable postharvest goals.	(12)
Red grapes	Plasma-activated water (PAW)	Chitosan-based coating with ascorbic acid (CH + AA)	PAW + CH + AA reduced microbes by ~2.62 log (bacteria), 1.72 log (yeasts/molds), 1.1 log (coliforms); boosted total phenols (~111.2 mg GAE/100 g) and flavonoids (~262.7 mg RE/100 g); inhibited water migration and cellular damage; preserved structure; extended shelf life by ≥ 8 days at 4 °C under vibration stress.	(43)

SI, synergistic index; CFU, colony-forming units; MAP, modified atmosphere packaging; WVP, water vapor permeability; PPO, polyphenol oxidase; POD, peroxidase; 4 °C, refrigerated storage temperature.

### 4.1 Evidence that they work well together and extend the shelf life

Researchers have discovered that combining plasma with edible coatings can greatly enhance the quality of fresh produce after harvest. For cherry tomatoes, the authors used plasma-activated water (PAW) as an antifungal and antibacterial agent, combined with a 5% Welsh onion leaf protein extract (WOLPE), creating plasma-activated WOLPE (PCW). PCW reduced bacterial and fungal populations on the surface of cherry tomatoes by 2.11 and 3.99 log CFU/g, respectively, while also delaying fruit softening, without negatively impacting color, pH, antioxidant capacity, sensory characteristics, or nutritional value. This treatment extended storage at 4 °C up to 14 days (43). The authors also paired PAW with a sodium caseinate guar gum, beeswax coating, which preserved physicochemical parameters, increased antioxidant activity, eliminated yeasts and molds, Enterobacteriaceae, and Pseudomonas bacteria, and suppressed total bacterial growth (about 2 log CFU/cm^2^) after 14 days at 4 °C (13).

In Granny Smith apples, applying cold plasma before the sodium alginate coating reduced weight loss and helped maintain firmness, titratable acidity, and color while decreasing microbial populations. Scanning electron microscopy images showed that cold plasma also improved the coating’s uniformity (11).

In another review, the authors confirmed that cold plasma activation can also enhance the mechanical strength and barrier properties of various edible coatings (12).

For red grapes, PAW was combined with a chitosan-based coating containing ascorbic acid (CH + AA) to reduce microbial counts to approximately 2–2.62 log for bacteria, 1.72 log for yeasts/molds, and 1.1 log for coliforms. Simultaneously, it increased total phenolic content (around 111.2 mg GAE/100 g) and flavonoid levels (about 262. 7 mg RE/100 g), inhibited water migration, reduced cellular damage, preserved structural integrity, and extended storage life by at least 8 days at 4 °C under vibration stress conditions (43).

In summary, these studies (Table 5) demonstrate that the synergy of plasma and edible coatings could be an innovative and sustainable approach to improving the safety, quality, and marketability of perishable produce. The reported outcomes can be interpreted within the framework shown in Figure 2, where most studies fall within the additive-to-synergistic transition zone.

### 4.2 Challenges and considerations for implementation

Although the combined PAW–EC strategy has shown significant benefits across various commodities, successful implementation depends on careful attention to process parameters, sequence, and compatibility between the two technologies. The following considerations are essential for translating laboratory findings into commercial applications.

The order of application is non-negotiable: PAW must precede EC application so that the antimicrobial RONS can act directly on the produce surface without being blocked by the coating barrier. A representative process flow is:

Receipt → batch PAW wash (5–10 min) → air drying → edible coating application → final drying → packaging.

The duration of the air-drying step between PAW treatment and coating application needs to be optimized. Residual surface moisture helps ensure even coating spreading and adhesion, but too much humidity can dilute the coating mixture and weaken the film. Finding specific drying windows for different commodities is an important factor for scale-up studies.

**Figure 3:**
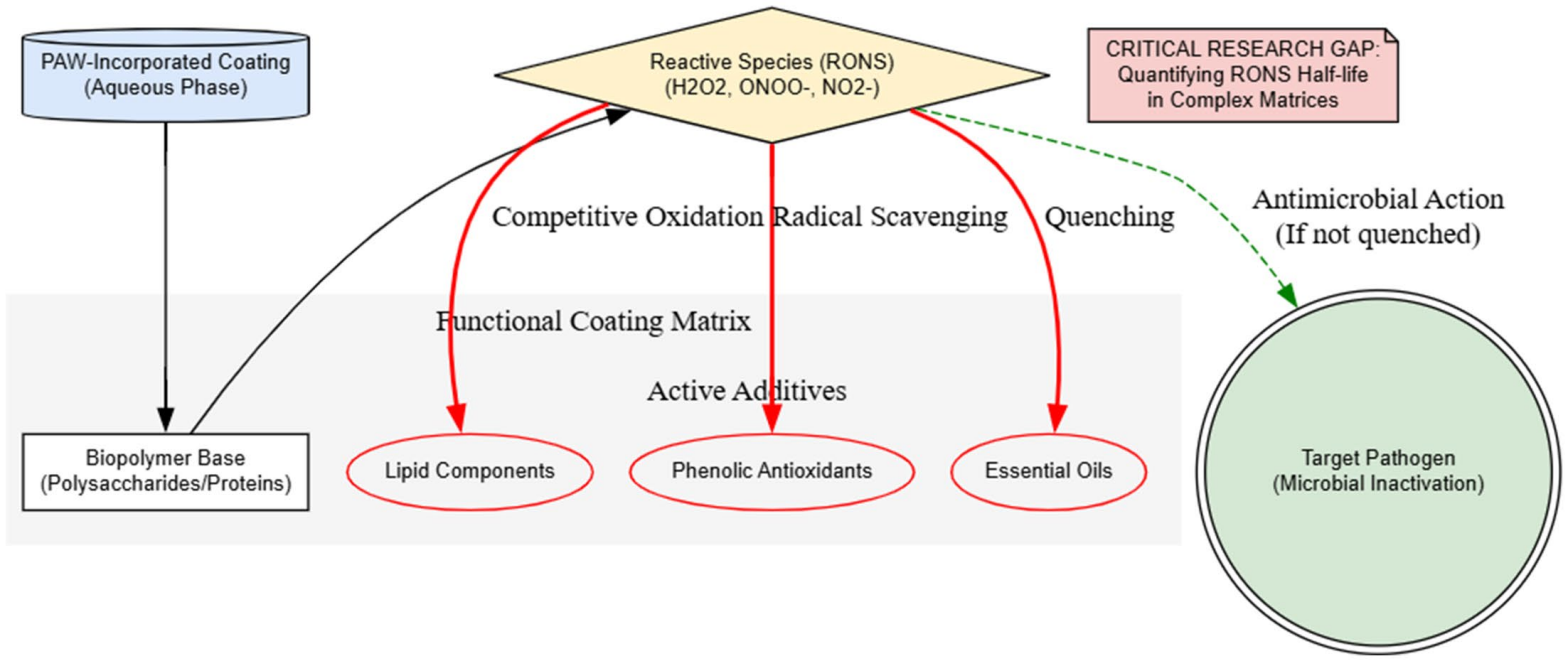
Conceptual framework of RONS interactions and quenching within PAW-incorporated edible coating systems. The figure illustrates the two competing mechanisms that determine the net outcome when PAW-modified produce surfaces are coated with an edible coating. The upper pathway (beneficial effect) shows that PAW-induced oxidation of the produce surface introduces polar functional groups—including hydroxyl (–OH), carbonyl (–C=O), and carboxyl (–COOH) groups—which reduce the water contact angle, increase surface energy, and enhance coating spreading and adhesion, leading to more uniform film formation. This surface modification has been confirmed by scanning electron microscopy in PAW-treated Granny Smith apples (11). The lower pathway (adverse effect) shows that reactive species remaining in the PAW, principally H_2_O_2_, are rapidly quenched upon contact with antioxidant additives (e.g., ascorbic acid, polyphenols), essential oil components, and lipid constituents within the coating formulation. This quenching can occur within seconds to minutes, depleting antimicrobial activity before RONS can diffuse to the produce-coating interface. High-viscosity polysaccharide and protein matrices further retard RONS diffusion. The net coating performance therefore depends on the balance between these competing effects, which varies with coating composition, application method, and produce commodity. RONS, reactive oxygen and nitrogen species.

PAW treatment modifies surface chemistry by increasing hydrophilicity and surface energy through oxidative reactions, a phenomenon well documented in materials science applications of plasma technology (12). This surface transformation and its implications for coating performance are conceptually illustrated in Figure 3. As shown in Figure 3, PAW-induced oxidation introduces polar functional groups, reducing the contact angle and enabling improved coating spreading and adhesion. These modifications may enhance coating wettability and adhesion, leading to more uniform film formation, as confirmed by scanning electron microscopy in Granny Smith apples (11). However, the extent to which PAW-induced surface changes improve or compromise specific coating formulations (e.g., high-viscosity polysaccharide gels vs. protein emulsions) remains to be systematically characterized across different commodity types.

A minimum hygiene standard must be maintained for coating solutions, especially when solutions are recirculated in industrial dip-tank systems, as recontamination from the solution itself could undermine the microbial reduction achieved by PAW. One emerging approach is to use PAW directly as the aqueous phase in coating formulations, thereby providing antimicrobial properties to the coating matrix. However, the stability of RONS in high-viscosity or emulsified matrices must be ensured, as reactive species may be rapidly quenched by lipid components or antioxidant additives in the formulation. Future research should focus on characterizing RONS half-lives in typical coating systems and on assessing whether residual antimicrobial activity persists after film drying and curing in high-viscosity systems. The variability in coating performance can be attributed to competing effects: improved adhesion from surface activation (Figure 3) and potential loss of antimicrobial efficacy from RONS quenching within coating matrices (Figure 3).

## 5 Sustainability and circular economy of edible coating technologies

### 5.1 Biopolymer sourcing and waste valorization

The main sustainability advantage of edible coatings comes from the source of their biopolymers. Unlike traditional petroleum-based packaging films, the ingredients of edible coatings (ECs), such as polysaccharides, proteins, and lipids, are obtained from renewable biological resources, including agricultural by-products, marine processing leftovers, and food manufacturing waste streams.^2^ Chitosan is made from chitin, which is sourced from crustacean shells, a major by-product of the seafood processing industry, with an estimated global annual production of 6–8 million tons. Pectin can be extracted from citrus peel waste produced by juice makers, alginate from seaweed biomass, and whey protein from dairy manufacturing byproducts. This connection with the valorization of industrial by-products is fundamental to circular bioeconomy principles, wherein waste from one process serves as a feedstock for another, thereby diminishing both raw material extraction and waste disposal burdens (10). Recent studies have shown that agro-industrial wastes, such as banana peels, pomegranate rinds, apple pomace, and sugarcane bagasse, can be used directly as functional coating components or as sources of bioactive chemicals, including polyphenolic antioxidants and antimicrobials. This method simultaneously addresses two inefficiencies in the food system: postharvest produce waste and the accumulation of food processing by-products (52). Cellulose-based coatings sourced from agricultural residues are compostable in ambient conditions, thereby preventing the creation of enduring environmental waste, in contrast to synthetic plastic packaging, which is anticipated to accumulate to 12,000 million metric tons globally by 2050 (53).

### 5.2 Life cycle sustainability and environmental footprint

From a life cycle perspective, edible coatings offer two main environmental benefits. First, they immediately reduce food waste, which is a significant source of greenhouse gas emissions, around 3.3 gigatons of CO₂ equivalent annually worldwide. They achieve this by extending the marketable shelf life of fresh produce by 40–100%. Every tonne of saved produce means less land, water, and energy are used in the supply chain. Second, because ECs are consumed with or break down on the surface of food, consumers do not need to discard secondary packaging, which reduces the solid waste cities must manage. The environmental profile of PAW is also positive. To produce PAW, only power, air, and water are needed; no chemicals or hazardous reagents are required. The reactive species it generates break down into harmless substances, such as water, oxygen, and trace nitrates, which are beneficial for farming. PAW is more environmentally friendly than chlorine-based sanitizers, which can leave long-lasting chlorinated disinfection by-products and require special storage and disposal procedures. Life cycle assessments of comparable cold plasma technologies consistently show they have less potential to cause global warming, harm aquatic life, and consume more energy than traditional chemical sanitation methods (12).

### 5.3 Alignment with UN sustainable development goals

The integrated EC–PAW platform directly supports several UN Sustainable Development Goals. Reducing postharvest losses helps SDG 2 (Zero Hunger) by increasing food supply without needing more agricultural land. Replacing synthetic chemicals with biodegradable, residue-free technologies promotes SDG 3 (Good Health and Wellbeing) by reducing consumer exposure to chemical preservative residues. Using agricultural by-products as coating feedstocks aligns with SDG 12 (Responsible Consumption and Production) by encouraging waste reduction and circular material use. Additionally, developing low-energy, low-waste preservation methods supports SDG 13 (Climate Action) by reducing greenhouse gas emissions associated with food loss (10).

A detailed bibliometric study by Ali et al. (10) demonstrated that research on edible coatings explicitly addresses SDG targets 2, 3, 8, 9, 12, 13, and 15, highlighting the cross-cutting sustainability importance of these technologies.

### 5.4 Circular economy outlook and future directions

The potential of EC technologies within the circular economy is expected to grow significantly as three key elements converge. Innovative green extraction methods, such as supercritical CO₂ extraction, ultrasound-assisted extraction, and enzymatic hydrolysis, are enabling the commercial-scale recovery of high-purity biopolymers from previously underused waste streams. Second, using PAW in coating formulations provides a dual-function system in which the coating itself has built-in antibacterial properties, eliminating the need for chemical preservatives. Third, the development of smart coatings with pH-sensitive or gas-responsive indicators made from natural anthocyanins or other plant pigments allows for real-time spoilage detection. This also helps reduce food waste by enabling consumers to intervene before discarding food out of caution. Regulatory frameworks in large markets are increasingly receptive to these approaches. In the U. S., the FDA categorizes most biopolymer coating materials as Generally Recognized as Safe (GRAS), and it is currently reviewing PAW as a processing aid under existing food contact regulations. In the European Union, adherence to the Farm to Fork Strategy and the EU Bioeconomy Strategy supports faster commercialization. To fully unlock the circular economy potential of EC–PAW systems, investing in scaling infrastructure, developing standardized life-cycle assessment methods for postharvest technologies, and fostering collaboration among food producers, biopolymer suppliers, equipment manufacturers, and regulators are crucial.

## 6 Conclusion and outlook

This review highlights that using plasma-activated water (PAW) followed by edible coating (EC) creates a well-founded, multi-layer preservation system. Its strength lies in its mechanistic complementarity. PAW provides rapid, broad-spectrum antimicrobial and anti-enzyme effects via RONS, while ECs provide continuous physical, chemical, and bioactive protection. Studies on strawberries, tomatoes, apples, and grapes demonstrate that combined treatments are more effective than individual methods, with microbial reductions of 2–4 log CFU/g, reduced enzymatic browning, and shelf-life extension of 40–100%. However, several key research gaps must be addressed before commercial application. Most studies are conducted in laboratory settings under optimized conditions; real-world testing under variable quality and environmental factors is still needed. The stability and activity of RONS when PAW is directly incorporated into coatings, especially in lipid-rich or thick matrices, require further investigation. Additionally, understanding how produce responds at the molecular level (via transcriptomics and metabolomics) remains limited, which impacts treatment optimization. From a regulatory and commercial perspective, both methods show promise. ECs made from GRAS biopolymers require minimal approval, and PAW, as a processing aid with no residues, could qualify for organic certification. Nonetheless, international standards for PAW characterization and RONS limits are necessary. Future research should focus on developing scalable PAW generators, creating commodity-specific coatings that utilize PAW’s surface adhesion properties, and incorporating active components such as spoilage indicators. Collaboration among scientists, engineers, regulators, and industry leaders is crucial to transforming research into large-scale applications that reduce processing time, maintain quality, extend shelf life, lower bacterial counts, and preserve color, firmness, and nutritional value. Efforts should also include developing rapid-drying systems and industrial-scale setups required for continuous PAW-EC processing lines.

## Footnotes

1 Cold plasma refers to ionized gas at near-room temperatures, suitable for treating heat-sensitive foods without thermal damage.

2 Edible coatings can be composed of polysaccharides, proteins, or lipids, depending on the functional and barrier properties required.

## Glossary

Plasma-Activated Water (PAW): Water treated with cold plasma, enriched with reactive oxygen and nitrogen species (RONS), used as a non-thermal antimicrobial rinse.
Edible Coatings (ECs): Thin, biodegradable layers applied to the surface of food to reduce moisture loss, improve shelf life, and carry active ingredients like antimicrobials or antioxidants.
RONS: Reactive Oxygen and Nitrogen Species generated during plasma treatment, including hydrogen peroxide (H₂O₂), nitric oxide (NO), and nitrate (NO₃^−^).
GRAS: “Generally Recognized as Safe” – a regulatory designation by the U. S. FDA for substances deemed safe for use in food.
Hurdle Technology: A preservation strategy combining multiple mild methods to control spoilage and pathogens without compromising food quality.
Clean Label: A consumer-driven food trend favoring products made with recognizable, natural, and minimal ingredients.
Transcriptomics/Metabolomics: Molecular techniques used to study gene expression (transcriptomics) and metabolite profiles (metabolomics) in biological samples.
